# A Metabolic Dependency for Host Isoprenoids in the Obligate Intracellular Pathogen Rickettsia parkeri Underlies a Sensitivity to the Statin Class of Host-Targeted Therapeutics

**DOI:** 10.1128/mSphere.00536-19

**Published:** 2019-11-13

**Authors:** Vida Ahyong, Charles A. Berdan, Thomas P. Burke, Daniel K. Nomura, Matthew D. Welch

**Affiliations:** a Department of Molecular and Cell Biology, University of California, Berkeley, Berkeley, California, USA; b Department of Nutritional Sciences and Toxicology, University of California, Berkeley, Berkeley, California, USA; University of Kentucky

**Keywords:** *Rickettsia*, isoprenoids, metabolic parasitism

## Abstract

Gram-negative bacteria in the order *Rickettsiales* have an obligate intracellular growth requirement, and some species cause human diseases such as typhus and spotted fever. The bacteria have evolved a dependence on essential nutrients and metabolites from the host cell as a consequence of extensive genome reduction. However, it remains largely unknown which nutrients they acquire and whether their metabolic dependency can be exploited therapeutically. Here, we describe a genetic rewiring of bacterial isoprenoid biosynthetic pathways in the *Rickettsiales* that has resulted from reductive genome evolution. Furthermore, we investigated whether the spotted fever group *Rickettsia* species Rickettsia parkeri scavenges isoprenoid precursors directly from the host. Using targeted mass spectrometry, we found that infection caused decreases in host isoprenoid products and concomitant increases in bacterial isoprenoid metabolites. Additionally, we report that treatment of infected cells with statins, which inhibit host isoprenoid synthesis, prohibited bacterial growth. We show that growth inhibition correlates with changes in bacterial size and shape that mimic those caused by antibiotics that inhibit peptidoglycan biosynthesis, suggesting that statins lead to an inhibition of cell wall synthesis. Altogether, our results describe a potential Achilles’ heel of obligate intracellular pathogens that can potentially be exploited with host-targeted therapeutics that interfere with metabolic pathways required for bacterial growth.

**IMPORTANCE** Obligate intracellular pathogens, which include viruses as well as certain bacteria and eukaryotes, are a subset of infectious microbes that are metabolically dependent on and unable to grow outside an infected host cell because they have lost or lack essential biosynthetic pathways. In this study, we describe a metabolic dependency of the bacterial pathogen Rickettsia parkeri on host isoprenoid molecules that are used in the biosynthesis of downstream products, including cholesterol, steroid hormones, and heme. Bacteria make products from isoprenoids, such as an essential lipid carrier for making the bacterial cell wall. We show that bacterial metabolic dependency can represent a potential Achilles’ heel and that inhibiting host isoprenoid biosynthesis with the FDA-approved statin class of drugs inhibits bacterial growth by interfering with the integrity of the cell wall. This work supports the potential to treat infections by obligate intracellular pathogens through inhibition of host biosynthetic pathways that are susceptible to parasitism.

## Abstract (precis)

Obligate intracellular pathogens, which include viruses as well as certain bacteria and eukaryotes, are a subset of infectious microbes that are metabolically dependent on and unable to grow outside an infected host cell because they have lost or lack essential biosynthetic pathways. In this study, we describe a metabolic dependency of the bacterial pathogen Rickettsia parkeri on host isoprenoid molecules that are used in the biosynthesis of downstream products, including cholesterol, steroid hormones, and heme. Bacteria make products from isoprenoids, such as an essential lipid carrier for making the bacterial cell wall. We show that bacterial metabolic dependency can represent a potential Achilles’ heel and that inhibiting host isoprenoid biosynthesis with the FDA-approved statin class of drugs inhibits bacterial growth by interfering with the integrity of the cell wall. This work supports the potential to treat infections by obligate intracellular pathogens through inhibition of host biosynthetic pathways that are susceptible to parasitism.

## INTRODUCTION

Gram-negative *Alphaproteobacteria* in the family *Rickettsiaceae*, which include the genera *Rickettsia* and *Orientia*, are obligate intracellular bacteria that can cause human diseases, including spotted fever (spotted fever group [SFG] *Rickettsia*), typhus (typhus group [TG] *Rickettsia*), and scrub typhus (*Orientia* species) (1). These bacteria are transmitted to mammals by arthropod vectors such as fleas, ticks, and mites. Although most pathogenic species cause moderately severe illnesses, in some cases infections can be fatal, even after treatment with first-line antibiotics (2). We study the SFG species Rickettsia parkeri, which causes an eschar at the site of the tick bite and symptoms that include fever, malaise, nausea, headaches, and an occasional rash (3, 4).

Upon invasion of host cells, *Rickettsia* species quickly escape the primary vacuole into the host cell cytoplasm, where the bacteria grow and proliferate. Obligate growth inside host cells has resulted in genome size reduction, and *Rickettsia* species have relatively small genomes of ∼1.1 to 1.5 Mbp (5–9) that encode a reduced number of proteins (1,273 predicted proteins in R. parkeri*;* NCBI reference sequence NC_017044.1). This typically correlates with the loss of genes encoding components of metabolic biosynthetic pathways, together with the requirement to scavenge essential metabolites from the host (5, 8).

**FIG 1:**
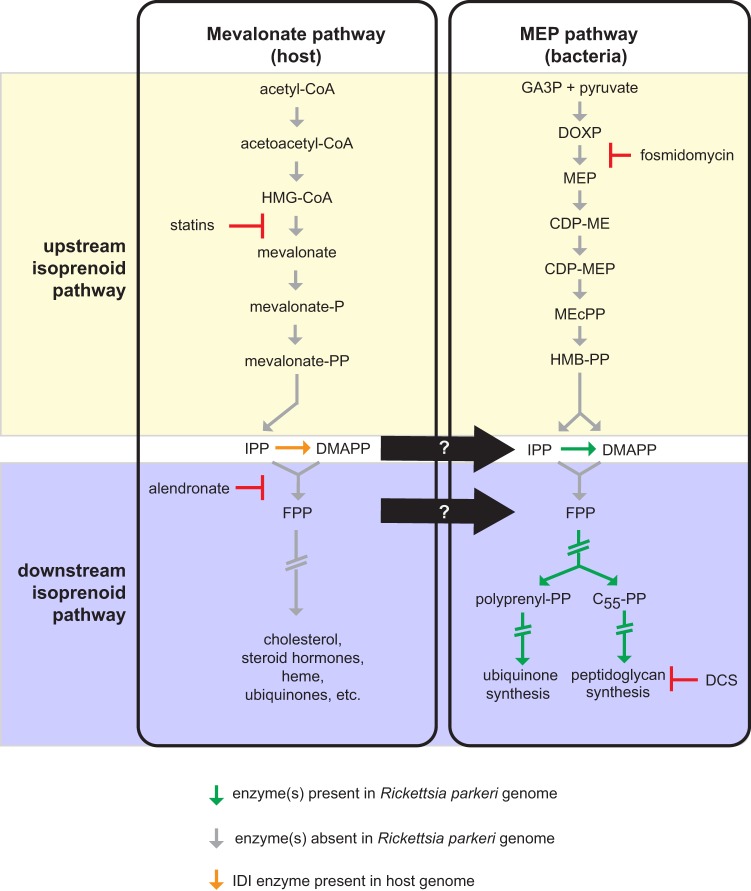
Generalized MEV and MEP pathways leading to downstream isoprenoid products. The green arrows indicate the presence of annotated enzymes encoded in the R. parkeri genome. The gray arrows indicate the absence of annotated enzymes encoded in the R. parkeri genome. The orange arrow indicates the presence of the IDI enzyme from the host genome. The yellow background denotes the upstream isoprenoid pathway, whereas the blue denotes the downstream isoprenoid pathway. The central black arrow and white question mark denote the possible presence of transporters for isoprenoids from the host into the bacteria. Arrows with double hash marks indicate multiple enzymatic steps. Statin drugs inhibit the activity of HMG-CoA reductase and the formation of mevalonate of the MEV pathway. Fosmidomycin is an inhibitor of DXP reductoisomerase of the MEP pathway. Alendronate sodium hydrate is an inhibitor of farnesyl diphosphate synthase. d-Cycloserine (DCS) is an inhibitor of d-alanine racemase and d-alanine-d-alanine ligase.

One essential class of metabolites are the isoprenoids (also known as terpenoids), which are derived from simple five-carbon isoprene units that are assembled to make thousands of different molecules. The biosynthesis of the central isoprene precursor molecule, isopentenyl pyrophosphate (IPP), and its isomer, dimethylallyl diphosphate (DMAPP), occurs through two distinct pathways: the mevalonate (MEV) pathway (predominantly in archaea, Gram-positive bacteria, and animals) or the 2-C-methyl-d-erythritol 4-phosphate (MEP) pathway (primarily in Gram-negative bacteria) (Fig. 1). In bacteria, isoprenoid biosynthesis produces vital products such as bactoprenols, which are essential building blocks for peptidoglycan (PG), chlorophylls in cyanobacteria, and the O-antigen of lipopolysaccharides (LPS), as well as ubiquinone and menaquinone, which are involved in the electron transport chain (10). In mammalian cells, isoprenoid biosynthesis produces a larger variety of products, including cholesterol, ubiquinone, steroid hormones, prenylated proteins, heme, and vitamin K (11).

Although the isoprenoid biosynthetic pathway is essential to most pathogens, its components are missing from certain obligate pathogens including *Mycoplasma* species (12) and the protozoan parasite *Cryptosporidium* (13). A recent bioinformatic analysis by Driscoll et al. (14) demonstrated that genes encoding the upstream enzymes from the MEV and MEP pathways that are required to make IPP and DMAPP are also missing in *Rickettsia* species, with the exception of the *idi* gene encoding the central isoprenoid pathway enzyme isopentenyl diphosphate isomerase (IDI), which catalyzes the reversible conversion of IPP to DMAPP (15). Furthermore, this analysis revealed that a *trans*,*trans*-farnesyl diphosphate (FPP) synthase involved in the synthesis of larger terpenoids, required for the generation of geranyl diphosphate (GPP) and FPP, is likewise missing within the *Rickettsia* species genomes (14). Because *Rickettsia* species have access to metabolites in the host cytoplasm, Driscoll et al. hypothesized that *Rickettsia* species may steal IPP, DMAPP, and FPP, altering the flux of host-derived isoprenoid precursors to initiate downstream bacterial isoprenoid biogenesis. The downstream enzymes required to utilize these short-chained isoprenoid precursors for ubiquinone biosynthesis and PG biosynthesis are encoded in *Rickettsia* species genomes.

In this study, we explore the evolutionary changes that occurred within the order *Rickettsiales* with respect to the presence of genes encoding components of the isoprenoid biosynthetic pathway. We also show that bacterial infection results in depletion of host isoprenoid products and the synthesis of bacterial isoprenoid products. Furthermore, we use a chemical genetic approach to reveal that inhibition of host isoprenoid biosynthesis by statin treatment prevents bacterial growth and alters bacterial shape. Our results suggest that the host MEV pathway serves as the upstream source of isoprene units for the synthesis of bacterial bactoprenols and ubiquinone and that inhibition of the host MEV pathway by statins may represent a promising avenue for host-directed therapeutics to limit *Rickettsia* species infection.

## RESULTS

### The isoprenoid biosynthesis pathway is under evolutionary flux in the order *Rickettsiales*.

**FIG 2:**
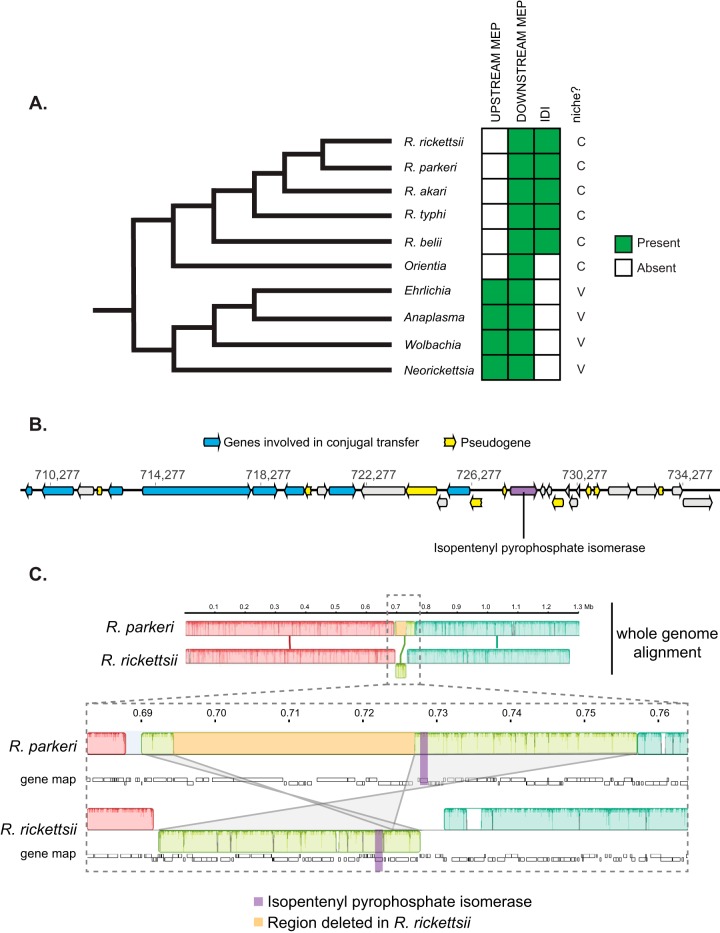
Genome evolution in the *Rickettsiales* isoprenoid pathway. (A) Schematic cladogram of the order *Rickettsiales*. The presence of intact upstream MEP pathway genes or of the *idi* gene for each species is indicated. The environmental niche is indicated on the right as follows: C, intracellular cytoplasmic; V, intracellular vacuolar. (B) A schematic of the R. parkeri *idi* genome locus. Genes involved in conjugal transfer and pseudogenes are indicated. The *idi* gene is highlighted in purple. (C) Whole-genome alignment of R. parkeri and R. rickettsii. The R. rickettsii genome has a large 33.5-kb deletion (in orange) in the genome alignment for the region adjacent to *idi*. The gray line segments indicate inverted alignment orientation between R. parkeri and R. rickettsii.

We first sought to determine the evolutionary conservation of the isoprenoid biosynthesis pathway in the order *Rickettsiales*. We analyzed six major genera of the order and assessed the presence or absence of genes encoding upstream or downstream components of the MEP pathway as well as the central isoprenoid pathway enzyme, IDI (Fig. 2A; see also Table S1 in the supplemental material). We confirmed that the *Rickettsia* (14) and *Orientia* lineages lack the genes encoding upstream MEP pathway enzymes but retain those encoding the downstream pathway components, whereas the other genera contained the full complement of MEP pathway genes. None have MEV pathway genes, but the *idi* gene is present in *Rickettsia* species. The absence of upstream MEP pathway genes suggests that *Rickettsia* species must scavenge MEV/MEP pathway intermediates (IPP, DMAPP and/or FPP) (Fig. 1) from the host cell.

10.1128/mSphere.00536-19.2 TABLE S1 Annotated R. parkeri isoprenoid genes. List of isoprenoid pathway genes and the enzymes they encode, with an indication of whether they are present or absent in the genome of R. parkeri. Download Table S1, PDF file, 0.03 MB. Copyright © 2019 Ahyong et al. 2019 Ahyong et al. This content is distributed under the terms of the Creative Commons Attribution 4.0 International license.

Additionally, the *idi* gene is present only in the *Rickettsia* lineage and not in the *Orientia* lineage. A previous report suggested that the *idi* gene in *Rickettsia* species was acquired by horizontal gene transfer (14). To further investigate this possibility, we examined the genomic locus surrounding the *idi* gene in the genome of the R. parkeri strain Portsmouth (Fig. 2B). We found many surrounding genes that were associated with conjugal transfer functions, such as transposases and pili. Furthermore, there are nine surrounding pseudogenes with premature stop codons, suggesting that this region is in flux and may be in the process of undergoing genomic reduction. To further test this hypothesis, we performed a whole-genome alignment between R. parkeri and the closely related yet more pathogenic species Rickettsia rickettsii. Interestingly, we found that the two genomes were highly syntenic, with the exception of a 65-kb region containing the *idi* gene (Fig. 2C, region in lime green and *idi* gene in purple) that is inverted between the two species and that in the R. rickettsii genome contains a 33.5-kb deletion (Fig. 2C orange highlighted region; Table S2). These observations suggest that the locus surrounding the *idi* gene was acquired by horizontal gene transfer (in agreement with previous phylogeny estimation [14] and with reports of similar genome rearrangements in *Rickettsia* species [16]) and is still under evolutionary pressure to retain the *idi* gene while simultaneously undergoing continuing reduction.

10.1128/mSphere.00536-19.3 TABLE S2 Genes missing in R. rickettsii 33.5-kb deleted locus. List of genes deleted in the 33.5-kb deleted region of the R. rickettsii genome but present in the R. parkeri genome. Also refer to Fig. 2C. Download Table S2, PDF file, 0.03 MB. Copyright © 2019 Ahyong et al. 2019 Ahyong et al. This content is distributed under the terms of the Creative Commons Attribution 4.0 International license.

### R. parkeri infection results in depletion of host isoprenoid products and accumulation of bacterial isoprenoid products.

**FIG 3:**
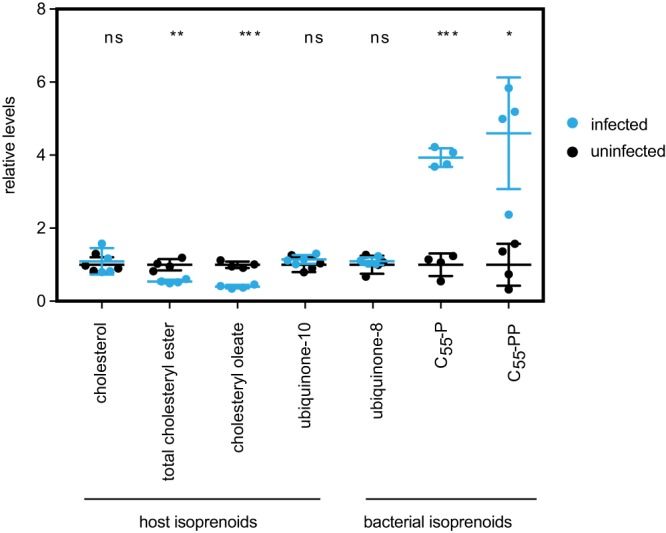
Targeted mass spectrometry of bacterial and host isoprenoids. Shown is a graph of the internal standard equivalent levels of host and bacterial isoprenoids at 4 dpi. Four technical replicates were done. Error bars represent standard deviations. Statistical comparisons were done by an unpaired Student's *t* test (ns, not significant; **, *P* < 0.01; ***, *P* < 0.001; ****, *P* < 0.0001, for results compared to those with the controls).

To test whether R. parkeri scavenges host isoprenoids to make bacterial products, we measured the presence and abundance of host and bacterial isoprenoid-derived metabolites by liquid chromatography tandem mass spectrometry (LC-MS/MS). Confluent infected and mock-infected cultures of African green monkey kidney epithelial Vero cell cultures were incubated for 4 days, collected, and then extracted for lipid metabolites. For host isoprenoids, we monitored cholesterol, total cholesteryl esters, cholesteryl oleate, and ubiquinone-10 using single-reaction monitoring (SRM)-based LC-MS/MS methods (Fig. 3). We observed a statistically significant, approximately 2-fold, decrease in total cholesteryl esters and cholesteryl oleate in the infected samples compared to levels in uninfected samples. These products are generally understood to be the storage units of cholesterol packaged in intracellular lipid droplets (17). In contrast, we saw no change in cholesterol or ubiquinone-10. Thus, infection results in the depletion of isoprenoid-derived storage forms of cholesterol.

For bacterial isoprenoids, we measured bactoprenols, including the isoprenoid products C_55_ pyrophosphate (C_55_-PP) and C_55_ phosphate (C_55_-P) (based on targeting for the [M-H]^−^ parent masses because there were no authentic standards available). C_55_-PP is initially produced by a dedicated prenyltransferase, UppS, and must be dephosphorylated to C_55_-P to act as a lipid carrier (18). We also measured bacterial ubiquinone, ubiquinone-8, which contains an 8-prenyl subunit tail, in contrast with the human version ubiquinone-10, which contains 10 prenyl subunits (Fig. 3) (19, 20). We observed an ∼4-fold increase in both C_55_-PP and C_55_-P in infected cells compared to levels in uninfected cells (values were normalized to internal standards [21, 22]). We did not, however, find significant differences in bacterial ubiquinone (ubiquinone-8) levels, possibly due to the presence of ubiquinone-8 intermediates from the host cell ubiquinone-10 biosynthesis pathway. These results suggest that bacterial isoprenoid products, primarily bactoprenols, accumulate during R. parkeri infection. Collectively, the depletion of host isoprenoid products and accumulation of bacterial isoprenoid products, even in the absence of a bacterial isoprenoid synthesis pathway, suggest that isoprenoids are scavenged from the host by R. parkeri.

### Chemical inhibition of the host mevalonate pathway inhibits bacterial growth.

**FIG 4:**
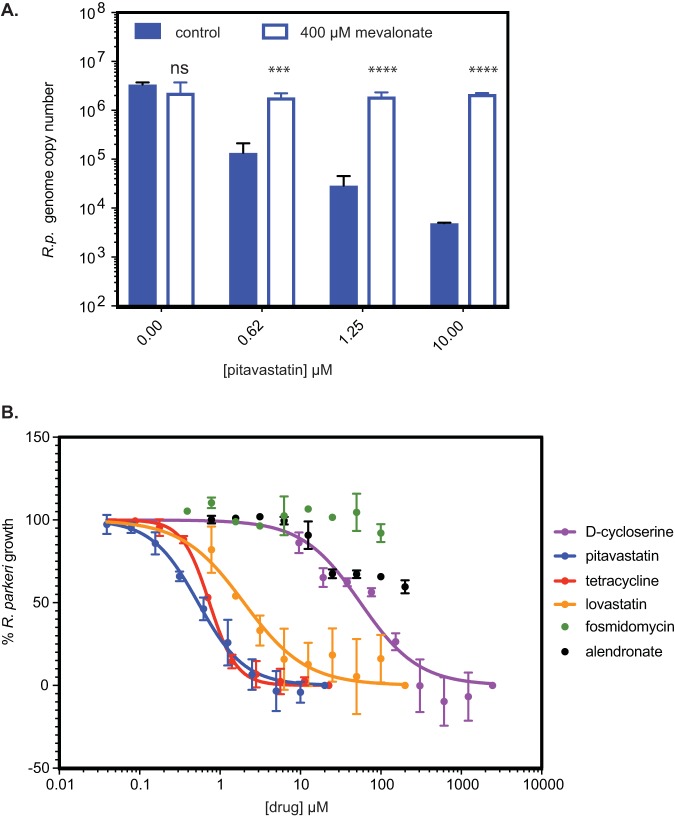
Chemical inhibition and rescue of R. parkeri growth. (A) Graph of R. parkeri genome copy numbers in the presence of various concentrations of pitavastatin, without or with mevalonate. Two independent biological replicates were performed, but four technical replicates from a single experiment are shown for simplicity. Error bars represent standard deviations. Statistical comparisons were done by an unpaired Student's *t* test for each concentration of pitavastatin for the wild type versus the wild type with mevalonate supplementation (ns, not significant; ***, *P* < 0.001; ****, *P* < 0.0001). R.p., R. parkeri. (B) Dose-dependent growth inhibition of R. parkeri in the presence of the indicated drugs targeting the MEV or MEP pathway enzymes or of tetracycline, normalized to that of a no-drug control. Fosmidomycin and alendronate failed to generate fit curves.

To determine whether host isoprenoids are necessary for bacterial growth, we sought to inhibit the host mevalonate pathway and reduce the pool of available host IPP, DMAPP, and FPP. We used statins, a class of drugs that block the activity of 3-hydroxy-3-methylglutaryl coenzyme A (HMG-CoA) reductase (Fig. 1). A previous report showed that preincubation of Rickettsia conorii-infected mouse L929 fibroblast cells with the statin lovastatin caused a reduction in R. conorii plaque size, which was interpreted to result from a reduction in cholesterol-dependent adherence of bacteria to host cells (23). To bypass possible effects of statins on bacterial adherence and invasion, we allowed infection of Vero cells to proceed for 2 h to allow for maximal bacterial adherence/invasion (24) and then, using untreated cells and cells treated with various concentrations of the statin pitavastatin, measured bacterial numbers using an R. parkeri*-*specific quantitative PCR (qPCR) endpoint assay (Fig. 4A). We observed a >2-log reduction in bacterial growth with increasing pitavastatin concentrations. To ensure that statin inhibition of bacterial growth was dependent on HMG-CoA reductase inhibition by pitavastatin and not on secondary effects of the drug, we tested whether the downstream product of HMG-CoA reductase, mevalonate, could rescue this growth inhibition. Indeed, cotreatment with 400 μM mevalonate and pitavastatin rescued bacterial growth (Fig. 4A).

To further assess the importance of the MEV pathway, we generated dose-response curves for the effects of lovastatin and pitavastatin, which are chemically distinct, on R. parkeri growth using a 96-well qPCR endpoint assay. We observed a dose-dependent inhibition of bacterial growth with lovastatin (50% effective concentration [EC_50_], 2.0 μM) and even more robust inhibition with pitavastatin (EC_50_, 0.5 μM) (Fig. 4B). The effect of statins on bacterial growth was not due to host cell death as treatment of uninfected cells throughout the dose-response range caused no significant loss of cell viability as measured by lactate dehydrogenase release (Fig. S1A) (although subcytotoxic concentrations of pitavastatin have been shown to alter cellular phenotypes such as the proportion of cells in different stages of the cell cycle, oxidant-induced apoptosis, or viability of different cancel cell lines [25–27]). Furthermore, host cells remained adherent throughout the dose-response range although there was an apparent decrease in cell-cell contact at concentrations of lovastatin and pitavastatin greater than the EC_50_ for bacterial growth inhibition (Fig. S1B). Together, these data indicate that the host MEV pathway is critical for R. parkeri growth.

10.1128/mSphere.00536-19.1 FIG S1 Host cell viability and monolayer integrity are maintained over the statin dose-response ranges. (A) Graphs show the mean percent LDH release for the indicated concentration of each drug. Data are from two independent experiments, each with three technical replicates. Error bars show the standard deviation. Statistical comparisons were done by one-way ANOVA of all drug-treated samples compared to a 1% Triton-X100-treated positive control sample; ns, not significant. (B) Bright-field images of Vero cell monolayers after 72 h incubation without statins or with the indicated concentration of statin. Download FIG S1, PDF file, 2.3 MB. Copyright © 2019 Ahyong et al. 2019 Ahyong et al. This content is distributed under the terms of the Creative Commons Attribution 4.0 International license.

To further test that bacterial growth is dependent on a complex integration of the host and pathogen isoprenoid pathways (depicted in Fig. 1), we tested for dose-dependent growth inhibition using additional inhibitors of MEV and MEP pathway enzymes (Fig. 4B). Fosmidomycin, which specifically inhibits 1-deoxy-d-xylulose 5-phosphate (DXP) reductoisomerase, a key enzyme in the bacterial MEP pathway, caused no inhibition of bacterial growth at up to 100 μM. Alendronate sodium, a specific inhibitor of host farnesyl diphosphate (FPP) synthase, caused approximately 50% growth inhibition. This further suggests that R. parkeri growth is independent of bacterial isoprenoid production and partially dependent on FPP from the host.

Finally, we tested the antibiotic tetracycline, which is used as a first-line treatment for *Rickettsia* species infection and targets protein synthesis, or d-cycloserine, which blocks the PG biosynthesis pathway that also requires isoprenoid biosynthesis products. Tetracycline inhibited growth, with an EC_50_ of 0.7 μM, consistent with MIC values for SFG rickettsiae of 0.06 to 0.25 μg/ml (0.1 to 0.5 μM) (28). d-Cycloserine also caused a dose-dependent inhibition of R. parkeri growth, with an EC_50_ of 56 μM, consistent with effects on Rickettsia prowazekii (29) and with MIC values for Escherichia coli K-12 (MIC of 30 μg/ml, or 290 μM) (30) and Mycobacterium tuberculosis (MIC of 15 μg/ml, or 150 μM) (31). Taken together, the results of chemical inhibition assays support the notion that *Rickettsia* species are dependent on the upstream host MEV pathway but not the upstream MEP pathway. Furthermore, they are susceptible to inhibition of PG biosynthesis.

### Statin treatment causes bacterial shape defects that mimic those caused by peptidoglycan-targeting antibiotics.

**FIG 5:**
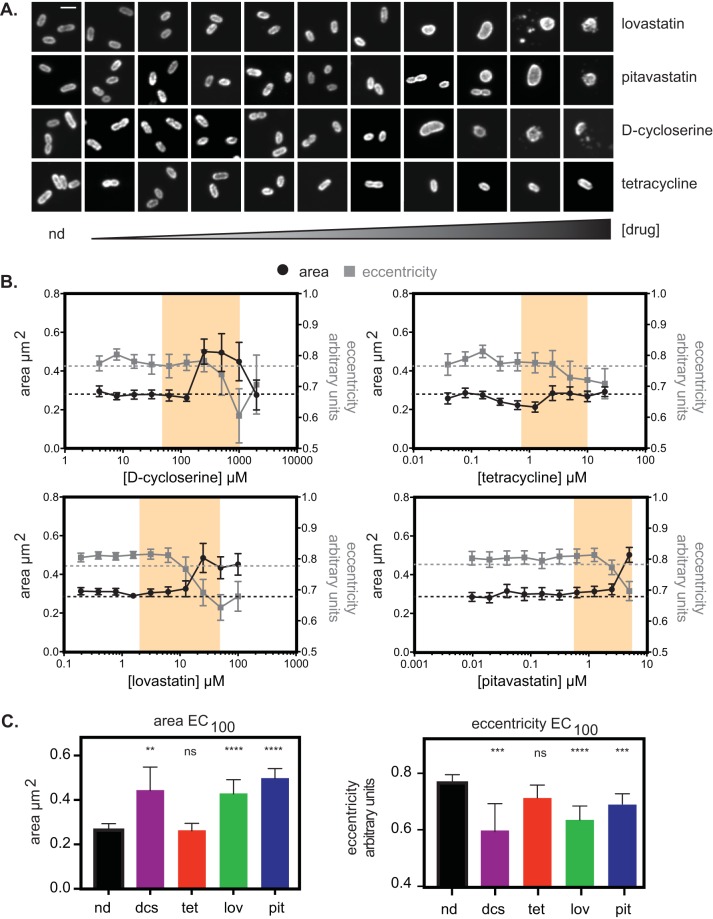
Shape and size measurements of R. parkeri bacteria under pressure from different drugs. (A) Representative bacterial cells from the EC curve drug concentrations from panel B. Scale bar, 1 μm. (B) Dose-dependent changes in area and eccentricity of R. parkeri bacterial cells under drug treatment with lovastatin, pitavastatin, tetracycline, or d-cycloserine. Each measurement is the mean of at least 40 individual bacterial cells, except for concentrations of d-cycloserine at >250 μM, where most bacterial cells were lysed and fewer measurements were possible (for 500 μM d-cycloserine, *n* = 22; for 1,000 μM d-cycloserine, *n* = 18; for 2,000 μM d-cycloserine, *n* = 13). Error bars represent the 95% confidence interval. (C) Graphs plotting area and eccentricity measurements at the EC_100_ values calculated from the data shown in Fig. 4B and matched to measurements from panel A. Regions shaded in yellow are the calculated values of EC_50_ to EC_100_ from the data shown in Fig. 4B. Graphs show the mean of the sample set, and error bars represent the 95% confidence interval. Statistical comparisons were done by one-way ANOVA of results for all drug-treated samples compared to results for the no-drug (nd) sample set (ns, not significant; **, *P* < 0.01; ***, *P* < 0.001; ****, *P* < 0.0001). dcs, d-cycloserine; tet, tetracycline; lov, lovastatin; pit, pitavastatin.

We sought to further assess the effect of statin treatment on bacterial physiology. Limiting the availability of isoprenoids is predicted to result in reduced PG synthesis. Though *Rickettsia* species (28), like other Gram-negative bacteria (32), are generally resistant to PG-targeted antibiotics, one study showed that high concentrations of penicillin produced spheroplasts (33), a type of L-form bacteria that have defective PG cell walls and take on a spherical shape (34). Therefore, we sought to measure whether statin inhibition of the host MEV pathway altered bacterial shape in a similar manner to inhibition of PG synthesis. To this end, we performed immunofluorescence microscopy on 96-well plates of infected cells at 3 days postinfection (dpi) to track the alterations of bacterial shape in response to dose-dependent application of statins, d-cycloserine, or tetracycline as a control. We performed automated image analysis using CellProfiler (35) and measured features of bacterial cell shape. We determined that two shape measurements, area and eccentricity (circle = 0; rod/line = 1), accounted for most of the shape alterations seen in the concentration ranges between the EC_50_ and EC_100_. In d-cycloserine-treated cells, there was a dose-dependent increase in area and decrease in eccentricity as bacteria became more circular and less rod shaped (Fig. 5A and B). In cells treated with either lovastatin or pitavastatin, similar changes in area and eccentricity were seen along the dose-response curve. In contrast, upon treatment with tetracycline, we found little change in the bacterial area although there was a general decrease in eccentricity.

Upon comparison of shape at the EC_100_ of each drug with that of the untreated control, we observed a significant increase in bacterial area and decrease in eccentricity in cells treated with d-cycloserine, lovastatin, and pitavastatin (Fig. 5C). For tetracycline, we found no significant differences in area or eccentricity compared to those of the untreated control group, likely because tetracycline does not directly interfere with cell wall biosynthesis. Thus, growth inhibition by statins causes bacterial shape defects that are similar to those caused by d-cycloserine, suggesting a shared mechanism of bacterial growth inhibition resulting from targeting host isoprenoid and bacterial PG synthesis pathways.

## DISCUSSION

Obligate intracellular pathogens such as *Rickettsia* species are dependent upon nutrients and metabolites from their host cells as a consequence of reductive genome evolution (5, 6, 14). This suggests the possibility that this metabolic dependency represents an Achilles’ heel in the host-pathogen relationship and that these pathogens could be sensitive to changes in host cell nutrient and metabolite levels. If metabolite availability is limited by the host or by extrinsic pressures, such as chemical inhibition of a host biosynthetic pathway, pathogen growth should also be reduced. Here, we report the reduction of R. parkeri replication when the host isoprenoid pathway is inhibited with statins. This growth inhibition correlates with changes in bacterial shape that are consistent with defects in cell wall biosynthesis. Our results suggest that statins interfere with *Rickettsia* species scavenging of host isoprenoids used for downstream biosynthetic pathways such as peptidoglycan biosynthesis, measured in this study.

We observed that the isoprenoid biosynthesis pathway is under evolutionary flux in the order *Rickettsiales*, with the *Rickettsia* and *Orientia* lineages having lost genes encoding the upstream components of the MEP pathway and *Rickettsia* species having gained the *idi* gene, consistent with previous findings (14). Through our understanding of reductive genome evolution and gene acquisition in prokaryotes (5, 6, 8, 36), we can surmise a most parsimonious order of loss and gain of isoprenoid biosynthetic pathway genes in the *Rickettsiales*. We propose that the common ancestor of the *Rickettsia* and *Orientia* lineages, which inhabit the host cell cytoplasm, may have gained the ability to scavenge IPP, DMAPP, and/or FPP from the cytoplasm by acquisition of a gene encoding a transporter that moves these highly charged pyrophosphate molecules into the bacterial cell. There is evidence for the presence of such transporters in plants, bacteria, and protozoan parasites although their molecular identities remain uncertain (37–39). Furthermore, previous studies in R. prowazekii have implicated metabolite transporters that allow the acquisition of triose phosphates from the host cell (40, 41). Gaining an IPP transporter would have enabled the loss of the upstream MEP pathway genes to reduce the bacterial genome and reduce fitness costs of pathway redundancy. Subsequently, the *idi* gene was gained in the *Rickettsia* lineage, which may reflect a need for additional DMAPP beyond that acquired from the host MEV pathway. In contrast, *Ehrlichia*, *Anaplasma*, *Wolbachia*, and *Neorickettsia* lineages, which grow within a membrane-bound vacuole, may not have sufficient access to host IPP and/or DMAPP and therefore have retained the entire MEP pathway.

We also found evidence of metabolic parasitism of host isoprenoids by R. parkeri. In particular, infected host cells became depleted of isoprenoid-derived storage forms of cholesterol, total cholesteryl esters, and choleteryl oleate, perhaps to balance free cholesterol levels. Curiously, we did not find differences in host or bacterial ubiquinone levels during infection, perhaps due to host compensation for reduced ubiquinone in response to infection. An additional complication of this metabolite analysis is the fact that ubiquinone-8 is an on-pathway intermediate in the synthesis of host ubiquinone-10, possibly masking our ability to distinguish changes in either host or bacterial ubiquinones in infected versus uninfected cells by mass spectrometry. Whether *Rickettsia* species also scavenge downstream host isoprenoid products such as cholesterol, as observed for other bacteria such as M. tuberculosis and Chlamydia trachomatis (42–44), remains to be determined. Furthermore, R. parkeri produces downstream isoprenoid products even though it lacks genes for the upstream components of the MEP pathway. This metabolic parasitism suggests that inhibition of host isoprenoid biosynthesis should reduce the ability of the bacteria to synthesize bacterial isoprenoid products.

In keeping with this prediction, we found that statins, which inhibit host HMG-CoA reductase, halt R. parkeri growth. Previous work had established an ability of statins to limit rickettsial plaque size (23), but the mechanism of inhibition was not explored in detail. It was suggested that statins might inhibit bacterial adherence to and/or invasion of host cells based on previous studies that had found a role for cholesterol in these processes (45, 46). Our work reveals an effect of statins on intracellular bacterial growth, downstream of adherence/invasion, as well as an effect on bacterial morphology that mirrors that which is caused by the cell wall synthesis inhibitor d-cycloserine. Whether statins have a bactericidal or bacteriostatic effect remains to be determined, but we observed morphological evidence of bacterial lysis at higher concentrations of statins, similar to lysis effects seen with d-cycloserine treatment but not with tetracycline treatment. These observations led us to conclude that statins inhibit bacterial growth by reducing host metabolite availability for production of bacterial products required for PG biosynthesis, leading to lethal defects in the bacterial cell wall. Future studies are also needed to determine if there are additional effects of statins on bacterial O-antigen and ubiquinone-8.

Whether statins can be effective at preventing or treating human *Rickettsia* species infections remains to be determined. Although statins are well tolerated in humans, we have limited understanding of the concentrations that would inhibit bacterial growth without causing toxicity. It is also unclear if statins cause the same degree of bacterial growth inhibition in different rickettsial target cell types *in vivo*. Additional studies in animal models will shed light on the future promise of statins as a prophylaxis or treatment for rickettsial infections. Currently, cases of rickettsial diseases are effectively treated with tetracyclines although there are reported cases of tetracycline-resistant scrub typhus (47, 48), and tetracycline treatment is contraindicated for pregnant women and young children (49). Statins are also contraindicated for pregnant women although in children (50) pitavastatin has been found to be both efficacious and safe (51). Because a limited number of antibiotics are effective in treating rickettsial infections (28), host-targeted therapeutics would be useful additions to treatment regimens. Furthermore, targeting host biosynthesis of essential metabolites may limit the development of antibiotic resistance.

Beyond the scope of rickettsial infections, this study supports the hypothesis of using host-targeted therapeutics for a wide variety of infectious microbes, including viruses, bacteria, fungi, and parasites. Similar to R. parkeri, the parasite Cryptosporidium parvum lacks the canonical protozoan isoprenoid pathway and has been shown to be sensitive to statins (52). Interestingly, the facultative intracellular protozoan parasite Toxoplasma gondii, which encodes the MEP isoprenoid pathway within a membrane-bound organelle called the apicoplast, has been shown to be sensitive to statins when the parasite is intracellular (53, 54), suggesting that host metabolite scavenging is still advantageous for pathogens with intact pathways. In the future, bioinformatic analyses may identify other intracellular bacteria that have incomplete upstream isoprenoid pathways and intact downstream isoprenoid pathways and thus may also be sensitive to statins. Furthermore, *Rickettsia* species and other parasites may be sensitive to chemical inhibition of other host metabolic pathways. Layering our knowledge of FDA-approved compounds that target host biosynthetic pathways onto predicted metabolic pathways hijacked by pathogens may therefore enable the systematic identification of drug classes that could be repurposed as antibiotics.

## MATERIALS AND METHODS

### Reagents.

Hoechst 33342 (B2261), d-cycloserine (C6880), tetracycline (T7660), fosmidomycin (F8682), mevalonate (M4667), and alendronate sodium hydrate (A4978) were obtained from Sigma-Aldrich. ProLong Gold Antifade (P36930), goat anti-mouse Alexa-488 secondary antibody (R37120), 0.25% trypsin-EDTA-phenol red (25200056), and a Dynamo HS SYBR green qPCR kit (F-410L) were obtained from ThermoFisher Scientific. Pitavastatin (494210) and lovastatin InSolution (4381875) were from Fisher Scientific. Anti-*Rickettsia* 14-13 antibody was generously provided by Ted Hackstadt, NIH/NIAID Rocky Mountain Laboratories (55).

### Cell culture and R. parkeri infections.

Confluent low-passage-number African green monkey epithelial Vero cells were obtained from the University of California Berkeley Cell Culture Facility and grown at 37°C in 5% CO_2_ and in high-glucose (4.5 g/liter) Dulbecco’s modified Eagle’s medium (DMEM) (11965092; Gibco/Life Technologies) supplemented with 2% fetal bovine serum (FBS; Benchmark). R. parkeri strain Portsmouth was generously provided by Chris Paddock (Centers for Disease Control and Prevention). R. parkeri was propagated by infecting monolayers of Vero cells with wild-type R. parkeri at a multiplicity of infection (MOI) of 0.1 and growing them at 33°C in 5% CO_2_ in DMEM plus 2% FBS. Bacteria were purified from infected cells as described previously (56). Briefly, infected cells were lysed by Dounce homogenization in cold K-36 buffer (0.05 M KH_2_PO_4_, 0.05 M K_2_HPO_4_, pH 7, 100 mM KCl, 15 mM NaCl) to release bacteria; the lysate was overlaid onto 30% MD-76R (1317-07; Mallinckrodt, Inc. ), centrifuged at 58,300 × *g* for 20 min at 4°C in an SW-28 swinging bucket rotor, and resuspended in cold brain heart infusion (BHI) broth (237500; BD Difco) Aliquots of bacteria were immediately frozen at –80°C after purification, and each infection was initiated from a single thawed aliquot of bacteria.

### *Rickettsiales* pathway analyses, *idi* locus mapping, and whole-genome alignments.

Cladogram schema were manually drawn based on relative distances from a previous phylogeny study of small subunit rRNA genes from the order *Rickettsiales* (57). The presence/absence of the MEP pathway or *idi* gene was determined using the KEGG pathway database (http://www.kegg.jp/) and manual annotations of the terpenoid backbone biosynthesis pathway (http://www.kegg.jp/kegg-bin/show_pathway?map=map00900) for each bacterial species. Determination of intracellular niche was performed by manual literature curation. The *idi* locus map and annotations were examined using Geneious software, version 9.1.8, using the R. parkeri strain Portsmouth genome sequence (NCBI RefSeq NC_017044.1). Whole-genome alignments of R. parkeri strain Portsmouth and R. rickettsii strain Iowa (NCBI RefSeq NC_010263.3) were performed using the Mauve, version 2.3.1 (58), plug-in in Geneious software using the progressive Mauve algorithm with automatically calculated-minimum locally colinear block (LCBs) scores.

### Mass spectrometry.

Monolayers of uninfected control Vero cells or Vero cells infected with R. parkeri were plated in six-well plates as described above and were incubated for 4 days at a 33°C in 5% CO_2_. On the day of harvest, cells in each well were washed with 4 ml of 1× phosphate-buffered saline (PBS). One milliliter of fresh 1× PBS was added to each well and used to scrape the cells, samples were centrifuged at 1,000 × *g* for 5 min on a microcentrifuge, and the supernatant was aspirated from the cell pellet. Cell pellets were extracted in 2:1:1 chloroform-methanol-PBS solution with addition of 10 nM dodecyl glycerol internal standard, after which the organic phase was separated, collected, and dried down under N_2_ gas. The dried-down lipidome extract was then resuspended in 150 μl of chloroform and stored at −80°C until analysis. For mass spectrometry analysis, an aliquot of this sample solution was injected into an Agilent 6430 LC-MS/MS instrument and was analyzed by single-reaction monitoring (SRM)-based targeted LC-MS/MS. Metabolite separation was performed using reverse phase chromatography, using a Luna reverse phase C_5_ column (50 mm by 4.6 mm with 5-μm diameter particles; Phenomenex), using the following mobile phases: buffer A consisting of 95:5 water-methanol and buffer B consisting of 60:35:5 2-propanol–methanol–water, both with 0.1% formic acid and 50 mM ammonium formate additives. The flow rate began at 0.1 ml/min for 5 min, followed by a gradient starting at 0% buffer B and increasing linearly to 100% buffer B over the course of 40 min with a flow rate of 0.4 ml/min, followed by an isocratic gradient of 100% buffer B for 10 min before equilibration for 5 min at 0% buffer B with a flow rate of 0.4 ml/min. MS analysis was performed using electrospray ionization (ESI) with a drying gas temperature of 350°C, drying gas flow rate of 10 liters/min, nebulizer pressure of 35 lb/in^2^, capillary voltage of 3.0 kV, and fragmentor voltage of 100 V.

C_55_-P and C_55_-PP levels were measured based on targeting for the parent [M-H]^−^ mass since there were no authentic standards available. The retention times for these metabolites correspond with the expected retention times based on those of shorter-chain pyrophosphate metabolites. For cholesterol, cholesteryl ester totals, cholesteryl oleate, and ubiquinone-10/8, we used single-reaction monitoring (SRM)-based methods monitoring the MS1-to-MS2 transitions based on fragmentation of standards. C_55_-P and C_55_-PP were measured in negative ionization mode, whereas cholesterol, total cholesteryl esters, cholesteryl oleate, and ubiquinone-10/8 were measured in positive ionization mode. Representative metabolites were quantified by SRM of the transitions from precursor to product ions at associated collision energies and retention times. Data were analyzed using Agilent Qualitative Analysis software by calculating the area under the curve (21, 22), and values are in picomoles relative to the internal standard control equivalents.

### Microscopy.

To measure bacterial shape, Vero cells were plated at 50% confluence on 96-well glass-bottom plates in DMEM plus 2% FBS and allowed to settle overnight at 37°C in 5% CO_2_. The following day, cells were infected at an MOI of 0.1 of wild-type R. parkeri, centrifuged at 300 × *g* for 5 min, and incubated for 2 h at 33°C in 5% CO_2_. Medium containing bacteria was aspirated and replaced with medium with or without the appropriate concentration of drug (see above), and cells were further incubated for 72 h at 33°C in 5% CO_2_. Cells were fixed with 4% paraformaldehyde in 1× PBS for 20 min at room temperature, washed with 1× PBS, and permeabilized with 0.05% Triton X-100 in 1× PBS for 5 min. Cells were then stained for immunofluorescence with mouse anti-*Rickettsia* primary antibody 14-13 and goat anti-mouse Alexa-488 secondary antibody to stain bacteria and Hoechst 33342 to stain DNA. Infected cells were imaged on a Nikon Ti Eclipse microscope with a Yokogawa CSU-XI spinning disc confocal, 100× (1.4 numerical aperture [NA]) plan apo objective, a Clara Interline charge-coupled-device (CCD) camera, and MetaMorph software taking 0.15-μm *z*-slices across 5 μm in the *z*-plane. Maximum *z*-projections for each channel were made using ImageJ, and a custom pipeline was created in CellProfiler software (35) to identify individual bacteria in each image. The CellProfiler module MeasureObjectSizeShape was used to calculate size and shape parameters of each bacterium. Bright-field monolayer images were acquired using an Olympus IX71 microscope equipped with a 20× LUCPlanFLN objective and a Photometrics CoolSnap HQ camera and MicroManager software.

### R. parkeri qPCR growth and drug dose-response curves.

Confluent Vero cells grown in 96-well tissue culture plates were infected with R. parkeri at an MOI of 0.1 and incubated in DMEM plus 2% FBS with or without the appropriate concentration of drug (see above) for 72 h at 33°C in 5% CO_2_. To harvest cells at the appropriate time point, medium was aspirated, and cells were lifted with 50 μl of 0.25% trypsin-EDTA followed by incubation at 37°C for 5 min. Lifted cells were resuspended with an additional 50 μl of DMEM before being added to 50 μl of Nuclei Lysis Solution (Wizard Genomic DNA Purification kit; Promega) and frozen at −20°C overnight. Cells were then thawed and boiled for 10 min to release genomic DNA. To remove RNA, 20 μg/ml RNase A was added to each sample, and samples were incubated for 15 min at 37°C and then cooled to room temperature. Protein was removed by addition of 50 μl of protein precipitation solution, mixed by pipetting, and then centrifuged in a microcentrifuge for 15 min at 1,500 × *g* at 4°C. DNA was precipitated by addition of 100 μl of the resulting supernatant to 100 μl of isopropanol, mixed by pipetting, and centrifuged for 15 min at 1,500 × *g* at 4°C. Isopropanol was removed, and the DNA pellet was washed with 70% ethanol and centrifuged for 15 min at 1,500 × *g*. Resulting DNA pellets were dried, resuspended in 50 μl of H_2_O, and allowed to rehydrate overnight at 4°C. For quantitative real-time PCR, 5 μl of genomic DNA was used with primers to the R. parkeri gene encoding the 17-kDa antigen (59), and runs were carried out on a Bio-Rad CFX96 Touch real-time PCR detection system. *Rickettsia* genome copy number was quantified against a standard curve of a plasmid containing a single copy of the R. parkeri 17-kDa gene. Regression analysis and EC_50_ calculations were performed using GraphPad Prism, version 7, software.

### LDH release assays.

For lactate dehydrogenase (LDH) release assays, Vero cells were plated and grown to confluence in a 96-well plate in 100 μl of DMEM (Gibco) containing 2% FBS (GemCell). The following day, medium was aspirated and replaced with 100 μl of fresh medium containing pitavastatin or lovastatin and incubated at 33°C. After 72 h, 60 μl of supernatant from wells containing Vero cells was collected into 96-well plates and 60 μl of LDH buffer (3 μl of diaphorase [DIA] solution containing 13.5 units/ml DIA [D5540; Sigma], 3 mg/ml β-nicotinamide adenine dinucleotide hydrate [N3014; Sigma], 0.03% bovine serum albumin [BSA], and 1.2% sucrose; 3 μl of INT solution, containing 2 mg/ml tetrazolium salt [I8377; Sigma] in PBS; 34 μl of PBS with 0.5% BSA; and 20 μl of solution containing 36 mg/ml lithium lactate in 10 mM Tris-HCl, pH 8.5 [L2250; Sigma ]) was added to each well. Supernatants from untreated cells and from cells lysed with 1% Triton X-100 were used as controls. Reaction mixtures were incubated at room temperature for 20 min prior to reading the absorbance at 490 nm using an Infinite F200 Pro plate reader (Tecan). Values for untreated Vero cells were subtracted from the experimental values, divided by the difference of Triton-lysed and untreated cells, and multiplied by 100 to obtain percent lysis. Each experiment was performed, and data were averaged between biological triplicates.

### Statistical analyses.

Statistical analysis was performed in GraphPad Prism, version 7, and statistical parameters and significance are reported in the figures and figure legends. Statistical significance was determined either by an unpaired Student's *t* test or a one-way analysis of variance (ANOVA) where indicated in the figure legends.

### Data availability.

Raw mass spectrometry data can be accessed at https://www.ebi.ac.uk/metabolights/MTBLS846.
