# Erratum for Ahyong et al., “A Metabolic Dependency for Host Isoprenoids in the Obligate Intracellular Pathogen Rickettsia parkeri Underlies a Sensitivity to the Statin Class of Host-Targeted Therapeutics”

**DOI:** 10.1128/mSphere.00848-19

**Published:** 2019-11-27

**Authors:** Vida Ahyong, Charles A. Berdan, Thomas P. Burke, Daniel K. Nomura, Matthew D. Welch

**Affiliations:** a Department of Molecular and Cell Biology, University of California, Berkeley, Berkeley, California, USA; b Department of Nutritional Sciences and Toxicology, University of California, Berkeley, Berkeley, California, USA

## ERRATUM

Volume 4, no. 6, e00536-19, 2019, https://doi.org/10.1128/mSphere.00536-19. Fig. 3: An incorrect version of this figure appeared in the published article. The correct figure is shown below.

**FIG 3:**
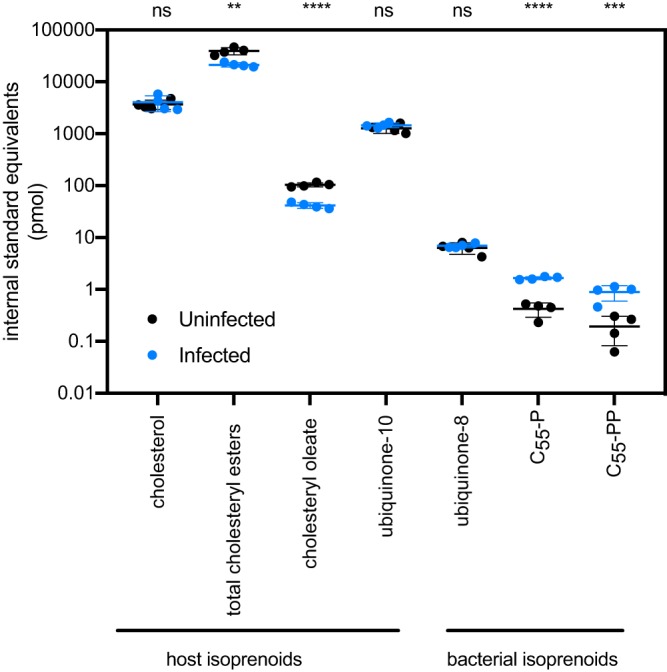
Targeted mass spectrometry of bacterial and host isoprenoids. Shown is a graph of the internal standard equivalent levels of host and bacterial isoprenoids at 4 dpi. Four technical replicates were done. Error bars represent standard deviations. Statistical comparisons were done by an unpaired Student’s *t* test (ns, not significant; **, *P* < 0.01; ***, *P* < 0.001; ****, *P* < 0.0001, for results compared to those with the controls).

