# Supplementary material for: A Metabolic Dependency for Host Isoprenoids in the Obligate Intracellular Pathogen Rickettsia parkeri Underlies a Sensitivity to the Statin Class of Host-Targeted Therapeutics
Source: mSphere. 2019 Nov 13;4(6):e00536-19. doi: 10.1128/mSphere.00536-19 (PMC6854040; doi:10.1128/mSphere.00536-19)
Supplement: TABLE S1 [file mSphere.00536-19-st001.pdf]

Supplemental Table S1: Annotated *R. parkeri* isoprenoid genes.

KEGG EC number	Gene Name	Upstream or Downstream Pathway	Present or Absent in R. parkeri	Enzyme Name
2.2.1.7	dxs	upstream	absent	1-deoxy-D-xylulose-5-phosphate synthase
1.1.1267	dxr	upstream	absent	1-deoxy-D-xylulose-5-phosphate reductoisomerase
2.7.7.60	ispD	upstream	absent	2-C-methyl-D-erythritol 4-phosphate cytidylyltransferase
2.7.1.148	ispE	upstream	absent	4-diphosphocytidyl-2-C-methyl-D-erythritol kinase
4.6.1.12	ispF	upstream	absent	2-C-methyl-D-erythritol 2,4-cyclodiphosphate synthase
1.17.7.1/1.17.7.3	gcpE/ispG	upstream	absent	(E)-4-hydroxy-3-methylbut-2-enyl-diphosphate synthase
1.17.7.4	ispH/lytB	upstream	absent	4-hydroxy-3-methylbut-2-en-1-yl diphosphate reductase
5.3.3.2	idi	central	present	isopentenyl-diphosphate Delta-isomerase
2.5.1.1	ggps	downstream	absent	geranylgeranyl diphosphate synthase
2.5.1.10	ispA	downstream	absent	farnesyl diphosphate synthase
2.5.1.90	ispB	downstream	present	octaprenyl-diphosphate synthase
2.5.1.31	uppS	downstream/PG	present	undecaprenyl diphosphate synthase
2.5.1.39	UbiA	downstream/Ubiquinone	present	4-hydroxybenzoate polyprenyltransferase
4.1.1.98	UbiD/UbiX	downstream/Ubiquinone	present	4-hydroxy-3-polyprenylbenzoate decarboxylase/flavin prenyltransferase
1.14.13240	UbiI	downstream/Ubiquinone	absent	2-polyprenylphenol 6-hydroxylase
2.1.1.222	UbiG	downstream/Ubiquinone	present	2-polyprenyl-6-hydroxyphenyl methylase / 3-demethylubiquinone-9 3-methyltransferase
1.14.13.-	UbiH/Coq6	downstream/Ubiquinone	present	2-octaprenyl-6-methoxyphenol hydroxylase
2.1.1.163/2.1.1.201	UbiE/Coq5	downstream/Ubiquinone	present	ubiquinone/menaquinone biosynthesis methyltransferase
1.14.99.60	UbiF/Coq7	downstream/Ubiquinone	present	3-demethoxyubiquinol 3-hydroxylase
2.1.1.222/2.1.1.64	UbiG/Coq3	downstream/Ubiquinone	present	bifunctional 3-demethylubiquinone-9 3-methyltransferase/2-octaprenyl-6-hydroxy phenol methylase
2.7.8.13	MraY	downstream/PG	present	phospho-N-acetylmuramoyl-pentapeptide-transferase
2.4.1.227	MurG	downstream/PG	present	undecaprenyldiphospho-muramoylpentapeptide beta-N-acetylglucosaminyltransferase
