# Supplementary material for: A Metabolic Dependency for Host Isoprenoids in the Obligate Intracellular Pathogen Rickettsia parkeri Underlies a Sensitivity to the Statin Class of Host-Targeted Therapeutics
Source: mSphere. 2019 Nov 13;4(6):e00536-19. doi: 10.1128/mSphere.00536-19 (PMC6854040; doi:10.1128/mSphere.00536-19)
Supplement: TABLE S2 [file mSphere.00536-19-st002.pdf]

Supplemental Table S2: Genes missing in *R. rickettsii* 33.5kb deleted locus

locus_tag	product	Min (original sequence)	Length	protein_id
MC1_RS03625	hypothetical protein	694497	1712	pseudogene
MC1_RS03630	DNA methyltransferase	696210	813	pseudogene
MC1_RS03635	sensor histidine kinase	697402	912	WP_014410747.1
MC1_RS03640	bifunctional (p)ppGpp synthetase/guanosine-3',5'-bis(diphosphate) 3'-pyrophosphohydrolase	698450	681	WP_014410748.1
MC1_RS03645	hypothetical protein	699600	2871	WP_014410749.1
MC1_RS03650	preprotein translocase subunit SecA	702483	6672	WP_014410750.1
MC1_RS03655	conjugal transfer protein TraD	709338	276	WP_041472287.1
MC1_RS03660	IS256 family transposase	709980	1216	pseudogene
MC1_RS07740	hypothetical protein	711323	646	pseudogene
MC1_RS07745	hypothetical protein	712056	219	pseudogene
MC1_RS07035	reverse transcriptase	712487	573	WP_014410755.1
MC1_RS03670	conjugal transfer protein TraA	713785	4137	WP_014410756.1
MC1_RS03675	transposase	717969	957	WP_041472215.1
MC1_RS03680	conjugal transfer protein TraD	719163	777	WP_081497779.1
MC1_RS07750	conjugal transfer protein TraD	719946	276	pseudogene
MC1_RS03685	hypothetical protein	720422	399	WP_014410759.1
MC1_RS03690	transposase	720858	1044	WP_014410760.1
MC1_RS03695	DUF87 domain-containing protein	722084	1695	WP_014410761.1
MC1_RS03700	hypothetical protein	723783	1195	pseudogene
MC1_RS07755	hypothetical protein	724964	408	WP_014410762.1
MC1_RS03705	F pilus assembly protein TraH	725338	894	WP_014410763.1
MC1_RS03710	conjugal transfer protein TraF	726221	462	pseudogene
