# Supplementary figures and images for: A Metabolic Dependency for Host Isoprenoids in the Obligate Intracellular Pathogen Rickettsia parkeri Underlies a Sensitivity to the Statin Class of Host-Targeted Therapeutics
Source: mSphere. 2019 Nov 13;4(6):e00536-19. doi: 10.1128/mSphere.00536-19 (PMC6854040; doi:10.1128/mSphere.00536-19)

**A.**

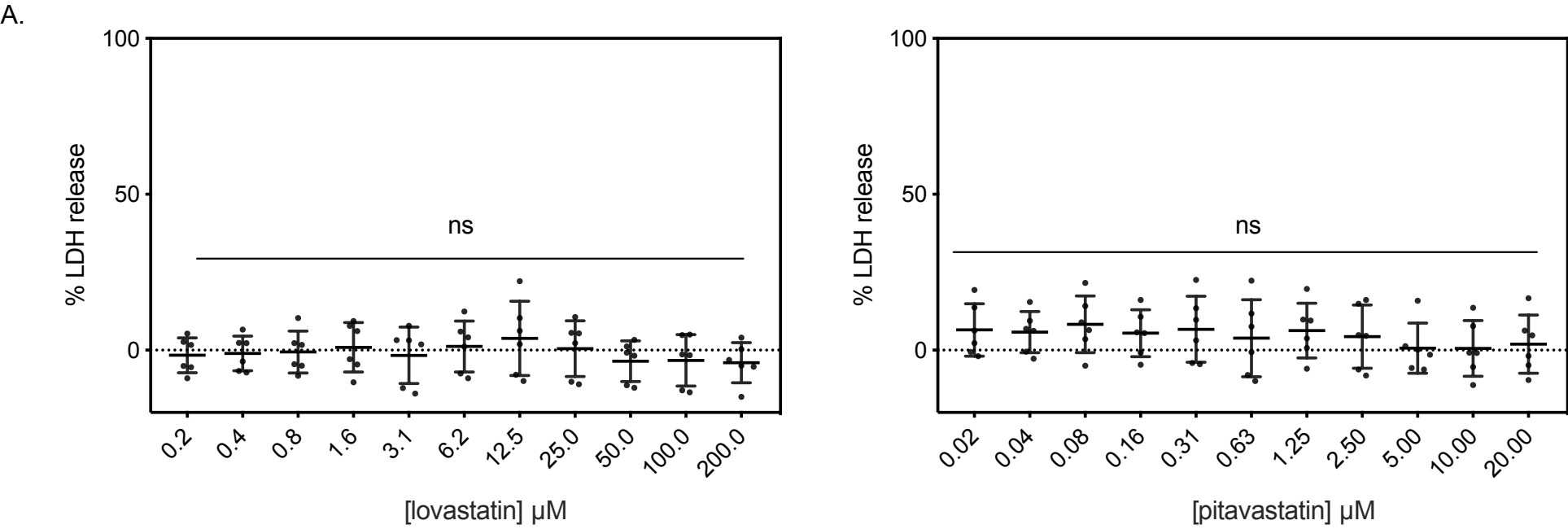

**B.**

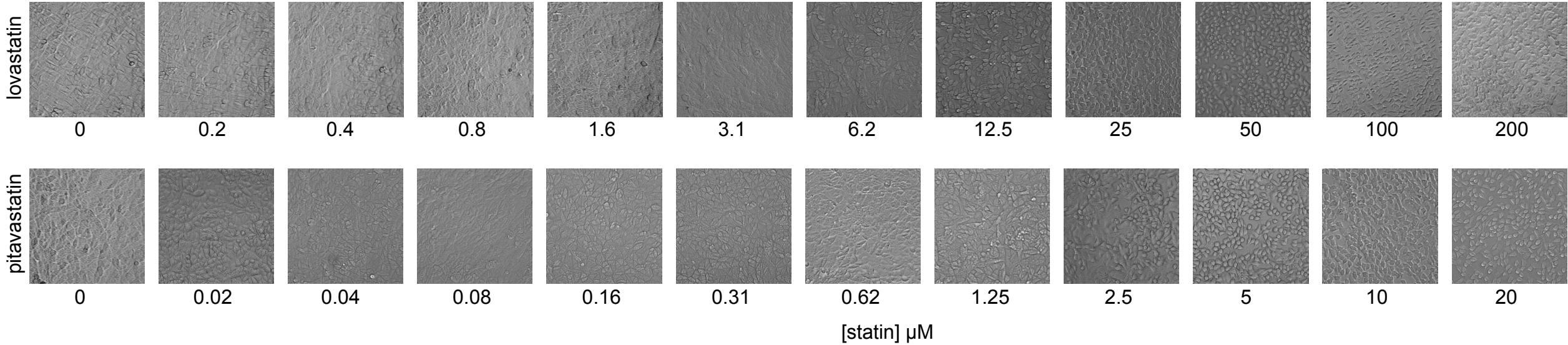

Supplement: FIG S1 [file mSphere.00536-19-sf001.pdf]
